# Phytotoxicity and cytogenotoxicity screening of cerium in contrasting tropical soils using rapid Petri-dish assays

**DOI:** 10.1007/s10653-026-03073-3

**Published:** 2026-02-23

**Authors:** Ingrid Fernanda Santana Alvarenga, Thaisa Aparecida Resende Pereira, Luiz Roberto Guimarães Guilherme, Larissa Fonseca Andrade-Vieira

**Affiliations:** 1 Departament of Biology, Federal University of Lavras, Lavras, MG 37200-900 Brazil; 2 Departament of Soil Science, Federal University of Lavras, Lavras, MG 37290-000 Brazil; 3 Departament of Ecology and Conservation, Federal University of Lavras, Lavras, MG 37290-000 Brazil

**Keywords:** Environmental risk assessment, Rare earth elements, Species sensitivity distribution (SSD), Cytogenotoxicity, Hazardous concentration (HC_5_)

## Abstract

Cerium (Ce), a rare earth element, can accumulate in agricultural soils through inputs such as phosphate fertilisers, yet effect thresholds for tropical soils remain scarce. Here, we performed a rapid plant-based screening of Ce toxicity in two representative Brazilian tropical soils (Latosol and Cambisol) and an artificial tropical soil (ATS). Seeds of *Pennisetum glaucum*, *Phaseolus vulgaris*, *Lactuca sativa*, and *Allium cepa* were exposed for 96 h to increasing Ce doses (0–2051.7 mg kg⁻^1^) in Petri-dish soil assays. These doses are reported as nominal (spiked) concentrations (post-equilibration concentrations were not measured). Macroscopic endpoints (germination, germination speed index, root and hypocotyl length, and fresh mass) were assessed for all species, and cytogenetic endpoints (mitotic index and nuclear/cytogenetic alterations) were evaluated in *A. cepa* root tips. Germination-related variables were generally less sensitive than seedling growth. Across endpoints, *L. sativa* showed the highest sensitivity, and effects were stronger in the Latosol compared with the Cambisol and ATS, indicating soil-dependent toxicity. EC_25_/EC_50_ values were obtained from concentration–response modeling, and a screening-level hazardous concentration for 5% of species (HC_5_) was derived from a species sensitivity distribution using EC_50_ values, resulting in HC_5_ = 208.4 mg Ce kg^−1^. This work provides a rapid screening framework and preliminary effect thresholds for Ce in tropical soils, while acknowledging limitations related to short-term exposure and the restricted set of test species/endpoints.

**Supplementary Information:**

The online version contains supplementary material available at 10.1007/s10653-026-03073-3.

## Introduction

Rare earth elements (REEs) have become an increasing source of interest for several segments of industry and agriculture (Barbieri et al., 2013; Feitosa et al., 2020) since they have ductile, malleable, resistant, and luminescent characteristics (Izatt et al., 2014; Jorjani & Shahbazi, 2012). Among the REEs, cerium (Ce) stands out for being considered the most abundant, representing 0.0043% of the Earth's crust (D'Aquino et al., 2009). This element can have positive effects at low concentrations in plants or harmful effects at high levels (Espindola et al., 2013; Hu et al., 2004; Thomas et al., 2014), and synthesis evidence indicates that plant responses to Ce are frequently dose-dependent and may involve stress-related pathways (Agathokleous et al., 2022). However, its mechanisms of action are still not fully clear, and therefore, the importance of further studies has been recognised (Baderna et al., 2011).

 Ce is naturally present in soils worldwide and its concentrations may increase in different contexts depending on parent material, land use, and anthropogenic inputs. In addition to dissolved ionic forms, Ce exposure scenarios increasingly include particulate and nanoparticulate forms (e.g., CeO_2_), and evidence from the plant–soil continuum highlights that its fate, transport, and biological interactions are strongly influenced by soil properties and exposure conditions (Prakash et al., 2021). Recent synthesis also indicates that Ce effects in plants are frequently dose-dependent and may involve stress-related pathways, reinforcing the need for effect-based screening across contrasting soil matrices (Agathokleous et al., 2022).

In Brazil, there are no reports of direct REE application through REE-enriched fertilizers in agriculture; however, agricultural inputs derived from phosphate rocks may contain significant levels of such elements (Bispo et al., 2021; Ramos et al., 2016a; Ribeiro et al., 2021). Tropical soils in Brazil have low natural fertility and high P fixation (Lopes & Guilherme, 2016); thus, to meet the nutritional requirements of crops, phosphate fertilisers are intensely applied (Roy et al., 2016; Withers et al., 2018). This high rate of soil phosphate fertilisation may result in significant increases in the levels of this REE in the environment (Bispo et al., 2021).

Despite being one of the largest producers and suppliers of agricultural products worldwide (OECD/FAO, 2015), Brazil has produced few studies that provide guidance on hazardous concentration values for Ce in Brazilian soils (Ribeiro et al., 2021). Therefore, effect-based toxicity screening studies are needed to understand the environmental consequences of Ce in soils and to support mitigation and preventive measures. However, soil toxicity assessment is inherently challenging because contaminant effects depend not only on nominal concentrations but also on soil matrix controls (e.g., pH, sorption-related properties, and organic matter), which influence speciation, retention, and bioavailability, and may lead to soil-dependent toxicity patterns (Prakash et al., 2021).

Importantly, soil quality monitoring is still largely based on chemical measurements and guideline comparisons; however, chemical data alone may not capture matrix-driven bioavailability, mixture effects, or site-specific interactions that modulate biological hazard. For this reason, there is an increasing trend to integrate chemical characterisation with effect-based approaches using bioassays across different endpoints, biomarkers, and organism models. In contaminated soil assessments, standardised plant tests (e.g., OECD/ISO growth assays) are widely used to screen phytotoxicity using endpoints such as germination and early seedling development. In parallel, genotoxicity can be incorporated as an additional layer of evidence through cytogenetic biomarkers, which are widely applied in soil monitoring and biomonitoring frameworks. In this context, higher-plant assays such as *Allium cepa* and the Vicia micronucleus test are established models for detecting chromosomal damage and related cytogenetic alterations, complementing conventional phytotoxicity endpoints (Gallego; Olivero-Verbel, 2021; Cotelle et al., 2015). Moreover, soil contamination assessments have increasingly combined plant tests with animal model organisms representing different exposure routes and ecological functions—such as nematodes (e.g., *Caenorhabditis elegans*), earthworms, and collembolans—to better capture soil-specific hazards and, when relevant, remediation outcomes (Rivenbark et al., 2024).

Accordingly, plant-based bioassays are widely used and can be highly efficient for assessing the potential risk of environmental contamination (Andrade-vieira et al., 2014). In particular, plant cytogenetic/genotoxicity endpoints have long been employed in environmental monitoring frameworks (US EPA, 1980) and provide sensitive effect-based evidence of chromosomal damage induced by pollutants. Moreover, several studies indicate that plant bioassays can show meaningful agreement with responses reported in other cellular systems, supporting their relevance for hazard screening (dos Reis et al., 2017). From a practical perspective, these assays are generally low-cost, rapid, and do not require approval by ethical committees (Andrade-Vieira et al., 2014), and they are consistent with 21st-century toxicological guidelines emphasizing replacement and reduction of animal testing (Hartung, 2009).

Bioassays with plants designed to investigate cyto and genotoxic responses are commonly performed in Petri dishes, combining macroscopic endpoints (e.g., germination and early seedling development) with microscopic endpoints related to cell division (mitosis), cytogenetic (chromosomes) and nuclear alterations (Leme and Marin-Morales, 2009; Silveira et al., 2017; dos Santos et al., 2018). By contrast, standardized plant tests focused on macroscopic growth parameters, such as OECD 208 and ISO 11269-2 (ISO, 2013), are typically performed in pots under greenhouse conditions.

 However, greenhouse pot assays are generally not designed to incorporate microscopic cytogenetic evaluations, which require observing meristematic cells in the mitotic cycle to quantify chromosomal aberrations and micronuclei (Leme & Marin-Morales, 2009). Petri-dish assays, in turn, can be conducted with smaller soil volumes and shorter exposure periods, enabling rapid, resource-efficient screening of soil contamination scenarios. This approach has been proposed as an expedient tier to provide early warning evidence of toxic potential and to help prioritise soils, contaminants, and concentration ranges for subsequent higher-tier testing and risk evaluation, particularly when large-scale monitoring is required (Alvarenga Et Al., 2025).

In this context, this study aimed to conduct a screening-level assessment of Ce phytotoxicity and cytogenotoxicity in contrasting tropical soils using rapid Petri-dish soil assays. Four plant species (*Pennisetum glaucum*, *Phaseolus vulgaris*, *Lactuca sativa*, and *Allium cepa*) were exposed to increasing Ce doses in two representative Brazilian tropical soils (Latosol and Cambisol) and an artificial tropical soil (ATS). Macroscopic endpoints (germination and early seedling development) were evaluated across all species, and cytogenetic endpoints in *A. cepa* root tips were used as complementary biomarkers, including the mitotic index (MI), condensed nuclei (CN), and micronucleus frequency (MCN), assessed in meristematic and F1 cells. Concentration–response modelling was applied to derive EC_25_​/EC_50_​ values, and an SSD-based HC_5_ was estimated to provide a screening-level threshold to support the prioritisation of soils and concentration ranges for subsequent higher-tier testing and risk evaluation. We hypothesized that Ce toxicity would be strongly soil-dependent, reflecting matrix controls on bioavailability (e.g., pH- and sorption-related properties) and leading to distinct effect thresholds among soil types.

## Materials and methods

### Plant material

Seeds of *Lactuca sativa* L. var. Great Lakes—Americana (lettuce); *Allium cepa* L. var. baia pear-shaped (onion); *Pennisetum glaucum* L. (millet), all from Isla® seeds; and *Phaseolus vulgaris* L. cv. BRSMG Uai (bean) were used. The *L. sativa* L., *A. cepa* L. and *P. glaucum* L. seeds were all from the same lot for standardization purposes, and the seeds of *P. vulgaris* L. were provided by the Department of Genetics and Plant Breeding of at the Federal University of Lavras (UFLA).

### Soil preparation

Two typical tropical soil classes were used: typical dystrophic Red–Yellow Latosol (Brazilian Soil Classification System—SiBCS; approximately corresponding to a Ferralsol (WRB) / Oxisol (USDA Soil Taxonomy)), medium to moderate texture (Table 1); and typical to moderate dystrophic Haplic Cambisol Tb (SiBCS; approximately corresponding to a Cambisol (WRB) / Inceptisol (USDA Soil Taxonomy)) (Table 1); both of which are found throughout much of the Brazilian territory. Additionally, an ATS (fine sand, kaolinitic clay, and coconut fiber at a 7:2:1 dry weight ratio) was used, according to the guidelines of OECD 208 (OECD, 2006), which recommends using an artificial substrate; we used the ATS for the comparison with Brazilian soils.

**Table 1:** Physical and chemical properties of the soils tested

	pH	K (mg dm^−3^)	P (mg dm^−3^)	Ca (cmol_c_dm^−3^)	Mg (cmol_c_dm^−3^)	Al (cmol_c_dm^−3^)	CEC (cmol_c_dm^−3^)	TOC (%)	Clay (%)	Silt (%)	Sand (%)
Latosol	5.20	39.42	0.88	0.14	0.10	0.20	1.73	0.80	28	4	68
Cambisol	6.10	39.42	1.14	2.26	0.13	0.03	04.08	01.09	32	48	20
ATS	5.60	192.71	4.89	1.44	0.44	0.16	3.63	4.00	–	–	–

Latosol and Cambisol properties were obtained from prior soil characterization (Moreira et al., 2019, with modifications) and are reported here to describe the experimental matrices; ATS = artificial tropical soil (OECD 208; sand:kaolinitic clay:coconut fiber, 7:2:1, w/w). pH (H₂O, 1:25). Available P and K (Mehlich-1; mg dm⁻^3^). Exchangeable Ca^2^⁺, Mg^2^⁺ and Al^3^⁺ (1 mol L⁻^1^ KCl; cmol_c_ dm⁻^3^). Potential acidity (H + Al) extracted with 0.5 mol L⁻^1^ calcium acetate (pH 7.0) and used to compute CEC (cmol_c_ dm⁻^3^). Organic carbon (dichromate oxidation; g 100 g⁻^1^ or as reported in Moreira et al., 2019). Particle-size fractions are given as %

The Latosol was collected in Itumirim-MG, Brazil (21°17′08″S, 44°47′43″W), and the Cambisol was collected in Lavras-MG, Brazil (21°13′46″S, 44°59′17″W). Both soils were collected under a minimally disturbed tropical semideciduous forest. These soils were selected due to their representativeness throughout the Brazilian territory.

The soils were contaminated with increasing doses of Ce (cerium chloride heptahydrate, CeCl₃·7H₂O): 50.00, 85.00, 144.5, 245.7, 417.6, 709.9, 1206.9, and 2,051.7 mg kg^−1^, with these doses being multiples of 1.7 (US EPA 1996) and ranging from natural concentrations in Brazilian tropical soils (82 mg Ce kg⁻^1^) (RAMOS et al., 2016b) to concentrations found in contaminated areas. Contamination was achieved by adding aqueous spiking solutions prepared from CeCl₃·7H₂O to soil aliquots, using proportional solution volumes to reach the target nominal doses, followed by thorough manual homogenization to minimize localized concentration gradients. Soil moisture was adjusted and maintained at 60% of field capacity according to ISO 11269-1 (ISO, 2012) throughout the exposure period. Field capacity/water-holding capacity (FC/WHC) was determined following ISO 11269-1 and used to calculate the total volume of spiking solution plus distilled water added to each soil aliquot to reach 60% FC/WHC. Accordingly, proportional volumes of the Ce solutions were added to the soils, taking FC/WHC into account, and distilled water was used as needed to standardize moisture across treatments (Alvarenga et al., 2025). Reported Ce concentrations refer to nominal (spiked) soil doses; Ce concentrations in soils and ATS components prior to spiking and post-equilibration Ce concentrations in the contaminated substrates were not analytically verified. Likewise, the pH of the spiking solutions and post-spiking soil pH were not measured; these constraints are acknowledged when interpreting effect thresholds.

### Treatments

The experiment was conducted in a completely randomized design, with five replicates (9 cm Ø polyethylene Petri dish) per treatment. Each Petri dish was filled with 60 g of Latosol and Cambisol or 50 g of ATS, and the field capacity was maintained at 60% according to the ISO 11269-1 (ISO, 2012). For each species tested, 25 seeds were distributed in the soil. Petri dishes containing filter paper soaked in 3 mL of ultrapure water were used as internal controls (ICs). As a negative control (NC), the soils tested were considered clean without the addition of Ce. The Petri dishes were placed in a biochemical oxygen demand (BOD) oven at 24 °C without a photoperiod (Narwal et al., 2009; Pinheiro et al., 2014) for 96 h.

### Macroscopic tests

 Germination of the four seed species was evaluated by recording the number of germinated seeds (with the protrusion of the root) every 24 h up to a total of 96 h and calculated according to Maguire (1962). To calculate the germination speed index (GSI), daily counts of the germinated plants were performed using the formula proposed by Maguire (1962):

$$IVG = \frac{G1}{N1}+\frac{G2}{N2}+\dots +\frac{GN}{NN}$$

where G1, G2, and GN represent the number of seeds germinated on the nth day.

N1, N2, and NN represent the number of days in which G1, G2, and GN were evaluated.

At the end of the experiment, the lengths of the emitted roots and hypocotyl were measured with the aid of a digital calliper. Next, the seedlings were washed in distilled water, and the fresh matter (FM) of the species was determined by weighing them on a precision scale.

### Analysis of the cell cycle

A microscopic test was performed on *A. cepa* L. root tips. After 96 h of the experiment, the roots were collected and fixed in Carnoy solution (ethanol:acetic acid, 3:1 v/v) and stored at − 4 °C for at least 24 h. The roots underwent hydrolysis with 1 mol L⁻^1^ HCl at 60 °C in a water bath and were subsequently exposed to Schiff’s reagent in the dark for 1 h and 30 min. To assemble the slides, 2% acetic carmine was used. The slides were prepared according to Silveira et al. (2017) using the squash technique.

The cell cycle parameters were evaluated in meristematic cells and F1 cells, as described in Palmieri et al. (2016). Then, the mitotic index (MI) and condensed nuclei (CN) were calculated when possible, following Palmieri et al. (2014). The frequency of changes in the cell cycle during segregation or chromosomal interphase was evaluated for meristematic cells. For F1 cells, we recorded only the frequency of micronucleus (MCN) and CN.

One slide was prepared per Petri-dish replicate, totalling five slides per treatment (n = 5). For each slide, two root tips were used for slide preparation (squash technique). Approximately 1,000 cells were scored per slide for each region evaluated, totalling 5,000 meristematic cells or 5,000 F1 cells analysed per Ce concentration per soil. The slides were evaluated under an Olympus CX 41 optical microscope (Olympus Corporation, Tokyo, Japan) at 400 × magnification.

MI was calculated as the number of dividing cells (prophase + metaphase + anaphase + telophase) divided by the total number of observed cells × 100. CN frequency and MCN frequency were calculated as the number of cells presenting condensed nuclei or micronuclei, respectively, divided by the total number of observed cells × 100.

### Statistical analysis

Normality and homoscedasticity were assessed using the Shapiro–Wilk and Levene tests, respectively (α = 0.05), for each soil × species × endpoint dataset. When assumptions were met, parametric procedures were applied as described below. When deviations from normality and/or homoscedasticity occurred, inference relied primarily on concentration–response modeling and the corresponding effect estimates (ECx), as recommended by Cedergreen et al. (2005). Assumption-test outcomes for all endpoints, species, and soils are summarized in Supplementary Table S4.

Germination (%) and germination speed index (GSI) were analyzed using one-way ANOVA followed by Dunnett’s test (*p* < 0.05) to determine the no observed effect concentration (NOEC) and lowest observed effect concentration (LOEC) in comparison with the control treatment. For all other endpoints, Ce effective concentrations (ECx) were derived from concentration–response curves, yielding EC25 and EC50 estimates as described by Cedergreen et al. (2005).

Risk analysis was performed using EC50 values considered valid for all species grown in Latosol and Cambisol. Species sensitivity distributions (SSD) were fitted (Posthuma et al., 2002) to estimate the hazardous concentration for 5% of species (HC5), i.e., the concentration expected to affect 5% or less of the tested species.

To evaluate how including data from plants grown in artificial tropical soil influences HC5 derivation, the same SSD analysis was also performed using data from all three soils. In addition, HC5 derivation was compared using (i) macroscopic endpoints only and (ii) macroscopic plus microscopic (cytogenetic) endpoints.

## Results

### Assumption checks

Normality (Shapiro–Wilk) and homoscedasticity (Levene) were evaluated for each soil × species × endpoint dataset (α = 0.05). Overall, homoscedasticity was largely supported, whereas normality was rejected for a subset of datasets (particularly for germination-related endpoints). Therefore, concentration–response modeling (EC25/EC50) was maintained as the primary inferential framework across endpoints, and ANOVA followed by Dunnett’s test (NOEC/LOEC) was applied to germination/GSI as a screening comparison to the control. Full assumption-test outcomes are summarized in Supplementary Table S4.

### Effects on germination

Descriptive results for all Ce doses (mean ± SD, n = 5) and Dunnett comparisons versus the control are provided in Supplementary Table S1 for germination (%) and GSI. Table 2 summarizes the resulting NOEC/LOEC values derived from these comparisons. The increasing concentrations of Ce had no negative effect on the germination of *P. glaucum* and *P. vulgaris* cultivated in Latosol, Cambisol, and ATS. For *A. cepa* germination, the LOEC was only calculated in the Latosol, with a value of 417.6 mg Ce kg^−1^. In *L. sativa* cultivated in Latosol, the LOEC found for germination was 245.7 mg Ce kg^−1^; in the Cambisol, it was 1206.9 mg Ce kg^−1^; and in the ATS, the LOEC was not found (Table 2).

**Table 2:** No observed effect concentration (NOEC) and lowest observed effect concentration (LOEC) estimated for the percentage of seed germination (%G) and germination speed index (GSI) of *L. sativa*, *A. cepa*, *P. vulgaris*, and *P. glaucum* exposed to soils (Latosol, Cambisol, and artificial tropical soil (ATS) contaminated with cerium (Ce)

Species	Parameters	Soils	NOEC	LOEC
			––mg Ce kg^−1^ de solo––	
*L. sativa*	Germination	Latosol	144.5	245.7
		Cambisol	709.9	1206.9
		ATS	2051.7	–
	GVI	Latosol	144.5	245.7
		Cambisol	417.6	709.9
		ATS	2051.7	–
*A. cepa*	Germination	Latosol	245.7	417.6
		Cambisol	2051.7	–
		ATS	2051.7	–
	GVI	Latosol	417.6	709.9
		Cambisol	2051.7	–
		ATS	2051.7	–
*P. vulgaris*	Germination	Latosol	2051.7	–
		Cambisol	2051.7	–
		ATS	2051.7	–
	GVI	Latosol	2051.7	–
		Cambisol	2051.7	–
		ATS	2051.7	–
*P. glaucum*	Germination	Latosol	2051.7	–
		Cambisol	2051.7	–
		ATS	2051.7	–
	GVI	Latosol	2051.7	–
		Cambisol	2051.7	–
		ATS	2051.7	–

Note: NOEC/LOEC are discrete values derived from Dunnett’s test (p < 0.05) relative to the control; confidence limits are not applicable. The symbol “-” means that the data obtained did not allow an estimation of the LOEC

 For the GSI, Ce caused no statistically significant difference in *P. vulgaris* and *P. glaucum* when grown in Latosol, Cambisol, and ATS (Table 2). For *A. cepa* cultivated in Latosol, the LOEC for the GSI was 709.9 mg Ce kg^−1^; when cultivated in Cambisol and ATS, there was no significant difference in the LOEC for the GSI. In *L. sativa*, the LOEC doses for the GSI were 245.7 mg Ce kg^−1^ and 709.9 mg Ce kg^−1^ in the Latosol and Cambisol soils, respectively, and no results were found for the ATS (Table 2).

### Effects of Ce on initial seedling development

Treatment-level descriptive statistics for root and hypocotyl length are provided in Supplementary Table S2.

Exposure to Ce decreased the initial development of the hypocotyls and roots of the seedlings for all species tested. The EC_x_ values for the initial development were lower than those found for the %G and GSI values and varied between the species tested and soil types.

In the Latosol soil, the most sensitive species, which had the lowest EC_50_, was *L. sativa* (EC_50_ root 346.3 mg Ce kg^−1^ and EC_50_ hypocotyl 318.9 mg Ce kg^−1^), followed by *A. cepa*, *P. vulgaris*, and *P. glaucum*. In the same soil, the species with the lowest sensitivity was *P. glaucum,* with EC_50_ values for the roots at 995.0 mg Ce kg^−1^ and the hypocotyls at 737.3 mg Ce kg^−1^ (Table 3).

**Table 3:** Effective concentration that resulted in 50% inhibition (EC_50_) and effective concentration that resulted in 25% inhibition (EC_25_) estimated for the root and hypocotyl lengths of *L. sativa*, *A. cepa*, *P. vulgaris*, and *P. glaucum* exposed to soils (Latosol, Cambisol, and artificial tropical soil (ATS)) contaminated with cerium (Ce)

Species	Parameter	Soil	EC_50_	EC_25_
*L. sativa*	Root	Latosol	346.3 (325.3–369.3)	296.1 (274.1–318.1)
		Cambisol	649.4 (720.2–965.1)	450.5 (417.8–865.3)
		ATS	824.5 (428.5–920.4)	682.1 (245.1–584.8)
	hypocotyl	Latosol	318.9 (206.5–231.3)	267.9 (222.5–304.9)
		Cambisol	762.7 (530.2–946.6)	478.8 (312.2–522.0)
		ATS	839.2 (646–1023.2)	675.3 (436.4–854.5)
*A. cepa*	Root	Latosol	512.7 (353.0–713.4)	468.4 (257.9–661.5)
		Cambisol	601.7 (497,5–721.4)	568.4 (454.6–643.8)
		ATS	894.7 (673.2–1036.2)	728 (515.2–972.1)
	hypocotyl	Latosol	697 (404.9–813.4)	803.85 (623.1–1004.7)
		Cambisol	943.1 (734.4–1132.2)	857.9 (712.2–996.3)
		ATS	1020.8 (883.6–1321.6)	987.9 (220.7–734.2)
*P. vulgaris*	Root	Latosol	595.9 (327.5–752.8)	498.2 (379.3–587.3)
		Cambisol	1160.1 (1031.4–1321.6)	1081.5 (898.3–1086.4)
		ATS	1653.3 (1287.1–1854.2)	1214.5 (902.7–1376.4)
	hypocotyl	Latosol	705.5 (580.2–846.6)	676.7 (534.5–813.4)
		Cambisol	2183 (1945.2–2351.5)	1940 (1686.4–2231.6)
		ATS	2651.3 (2232.1–2821.8)	2458 (2131.2–1612.6)
*P. glaucum*	Root	Latosol	995.0 (834.4–1092.7)	765.4 (654.4–912.6)
		Cambisol	1286 (1151.4–1411.4)	995.4 (774.3–1143.2)
		ATS	1499.3 (1371.1–1751.6)	1094.9 (791.1–1206.4)
	hypocotyl	Latosol	737.3 (621.4–872.9)	613.1 (486.4–742.9)
		Cambisol	1222 (986.4–1511.4)	1018 (801.4–1197.6)
		ATS	2081 (1823.5–1262.3)	1823 (1681.4–2019.6)

95% confidence limit

The EC_50_ and EC_25_ values were higher in all species in the Cambisol soil than in those in the Latosol soil. The EC_50_ and EC_25_ values in the *L. sativa* roots were 649.4 mg Ce kg^−1^ and 450.5 mg Ce kg^−1^, respectively, and those for the hypocotyls were 762.7 mg Ce kg^−1^ and 478.8 mg Ce kg^−1^, respectively. The species least sensitive to Ce were cultivated in the Cambisol soil, and of the species, *P. vulgaris* had the highest EC_50_ and EC_25_ values, as shown in Table 3.

For the ATS, root and hypocotyl development was more sensitive in *L. sativa* than in the other species as this species had the lowest EC_50_ and EC_25_ values; *P. vulgaris* had the least affected roots and hypocotyls in the Cambisol soil (Table 3).

### Effects of Ce on fresh matter

 Treatment-level descriptive statistics for fresh matter are provided in Supplementary Table S2. Regarding the effect of the Ce concentrations on the seedling FM for the species cultivated in the Latosol, Cambisol, and ATS, it was not possible to calculate the EC_50_ and EC_25_ for some species (Table 4). *P. vulgaris* did not experience significant effects on its FM, and therefore, it was not possible to generate such data in the three cultivated soils. For *A. cepa* cultivated in the Latosol, the EC_50_ value relative to the FM was 2106.7 mg Ce kg^−1^, and the EC_25_ relative to the FM was 1479.4 mg Ce kg^−1^; therefore, no results were obtained (Table 4).

**Table 4:** Effective concentration that resulted in 50% inhibition (EC_50_) and effective concentration that resulted in 25% inhibition (EC_25_) estimated for the fresh matter of *L. sativa*, *A. cepa*, *P. vulgaris*, and *P. glaucum* seedlings exposed to soils (Latosol, Cambisol, and artificial tropical soil (ATS)) contaminated with cerium (Ce)

Species	Soil	EC_50_	EC_25_
*A. cepa*	Latosol	2106.7 (2034.3–2187.9)	1479.4 (1393.4–1537.9)
	Cambisol	- -	- -
	ATS	- -	- -
*P. vulgaris*	Latosol	- -	- -
	Cambisol	- -	- -
	ATS	- -	- -
*P. glaucum*	Latosol	932.0 (1423.4–1717.9)	702.5 (667.5–776.4)
	Cambisol	1986.0 (1912.2–2062.5)	1195.4 (1121.3–1238.5)
	ATS	2107.3 (1065.5–2153.2)	1549.6 (1489.5–1606.1)

95% confidence limit

- The data obtained did not allow an estimation of the EC_x_

For *P. glaucum*, the Latosol had the lowest EC_50_ and EC_25_ values at 932.0 mg Ce kg^−1^ and 702.5 mg Ce kg^−1^, respectively; the Cambisol had an EC_50_ of 1986.0 mg Ce kg^−1^ and an EC_25_ of 1195.4 mg Ce kg^−1^. The highest for EC_50_ and EC_25_ values occurred in the ATS at 2107.3 mg Ce kg^−1^ and 1549.6 mg Ce kg^−1^, respectively (Table 4).

### Changes in the cell cycle and interphase of meristematic cells

Treatment-level frequencies of cell-division phases and mitotic index (mean ± SD) are provided in Supplementary Table S3. The EC_25_ and EC_50_ values for the changes in the meristematic cells varied depending on soil type (Table 5), and these parameters were only evaluated in *A. cepa*. The Latosol generally had the lowest EC_x_ values generated from the cell cycle, followed by the Cambisol and ATS.

**Table 5:** Effective concentration that resulted in 50% inhibition (EC_50_) and effective concentration that resulted in 25% inhibition (EC_25_) estimated for the mitotic index (MI), chromosomal aberrations, condensed nuclei (CN), and micronuclei (MCN) of *A. cepa* roots exposed to soils (Latosol, Cambisol, and artificial tropical soil (ATS)) contaminated with cerium (Ce)

Parameters	Soil	EC_50_	EC_25_
MI	Latosol	1572.7 (1423.4–1717.9)	1411.5 (1232.4–1603.7)
	Cambisol	–	–
	ATS	–	–
Chromosomal Aberrations	Latosol	66.7 (54.4–81.7)	57.4 (42.9–72.7)
	Cambisol	253.0 (212.5–297.4)	389.4 (297.3–472.9)
	ATS	1236.9 (1172.5–1291.2)	583.7 (504.2–662.1)
CN	Latosol	930.6 (888.5–976.2)	542.7 (502.5–603.1)
	Cambisol	1104.9 (1022.2–1191.4)	1031.5 (978.5–1089.6)
	ATS	1314.5 (1279.5–1354.1)	1272.9 (1248.1–1307.9)
MCN	Latosol	338.4 (297.54–384.2)	114,9 (95.4–136.4)
	Cambisol	445.1 (413.2–479.6)	294.2 (251.2–332.5)
	ATS	683.7 (576.7–797.5)	595.3 (533.3–677.1)

95% confidence limit

Regarding the cell cycle analyses, for the MI in the Latosol, the dose for EC_50_ was 1572.7 mg kg^−1^, and that for EC _25_ was 1411.5 mg kg^−1^. In the Cambisol and ATS, it was not possible to calculate the EC_50_ and EC_25_ (Table 5).

 Regarding chromosomal aberrations (CAs), in the Latosol, the EC_50_ and EC_25_ values were 66.7 mg Ce kg^−1^ and 57.4 mg Ce kg^−1^, respectively. The Cambisol showed intermediate values, with an EC_50_ value of 253.0 mg Ce kg^−1^ and an EC_25_ value of 389.4 mg Ce kg^−1^. In the ATS, which had the highest CA values, the EC_50_ value obtained was 1236.9 mg Ce kg^−1^, and EC_25_ value was 583.7 mg Ce kg^−1^ (Table 5).

 Nuclear changes, such as in the CN and MCN, were found in the meristematic cells of all soils analyzed. The EC_50_ and EC_25_ values for the NC in the Latosol were 930.6 mg kg^−1^ and 542.7 mg kg^−1^, respectively, and those for the MCN were 338.4 mg kg^−1^ and 114.9 mg kg^−1^ (Table 5).

In the Cambisol soil, the EC_50_ and EC_25_ values for the NC were 1104.9 mg Ce kg^−1^ and 1031.5 mg Ce kg^−1^, respectively, and those for the MCN were 445.1 mg Ce kg^−1^ and 294.2 mg Ce kg^−1^. In the ATS, the EC_50_ and EC_25_ values for the NC were 1314.5 mg Ce kg^−1^ and 1272.9 mg Ce kg^−1^, respectively, and those for the MCN were 683.7 mg Ce kg^−1^ and 595.3 mg Ce kg^−1^ (Table 5).

### Micronuclei in the F1 region

In the F1 region, which is located above the meristematic region and has cells beginning the differentiation process, the presence of MCN was observed. It was possible to calculate the ecotoxicological indices for the three soils tested (Table 6).

**Table 6:** Effective concentration that resulted in 50% inhibition (EC_50_) and effective concentration that resulted in 25% inhibition (EC_25_) estimated for the micronucleus (MCN) of the F1 region of *A. cepa* roots exposed to soils (Latosol, Cambisol, and artificial tropical soil (ATS)) contaminated with cerium (Ce)

Species	Soil	EC_50_	EC_25_
MCN	Latosol	425.4 (359.3–505.6)	331.1 (287.5–391.9)
	Cambisol	982.5 (875.6–1094.2	528.6 (463.7–596.3)
	ATS	1196.2 (1072.5–1306.1)	887.1 (792.7–969.4)

95% confidence limit

 The MCN in the Latosol had the lowest EC_50_ and EC_25_ values at 425.4 mg kg^−1^ and 331.1 mg kg^−1^, respectively; these values were followed by those in the Cambisol at 982.5 mg kg^−1^ and 528.6 mg kg^−1^. The highest EC_50_ and EC_25_ values were obtained in the ATS at 1196.2 mg kg^−1^ and 887.1 mg kg^−1^, respectively (Table 6).

**Fig. 1:**
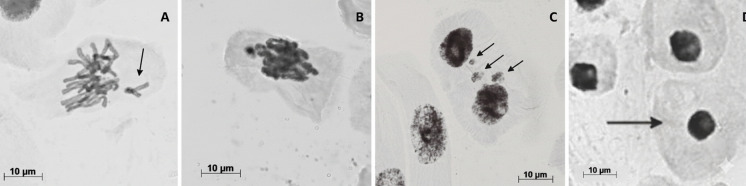
Representative cytogenetic and nuclear alterations in *Allium cepa* root tip cells after exposure to Ce-spiked soils: (**A**–**B**) chromosomal alterations (examples): non-oriented chromosome (**A**) and Stickness chromosomes (**B**), (**C**) micronuclei (MCN), and (**D**) condensed nucleus (CN). Scale bars = 10 µm

Representative photomicrographs illustrating the main chromosomal alterations (meristematic cells), condensed nuclei, and micronuclei (meristematic and F1 regions) are shown in Fig. 1.

### Ecotoxicological risk assessment

**Fig. 2:**
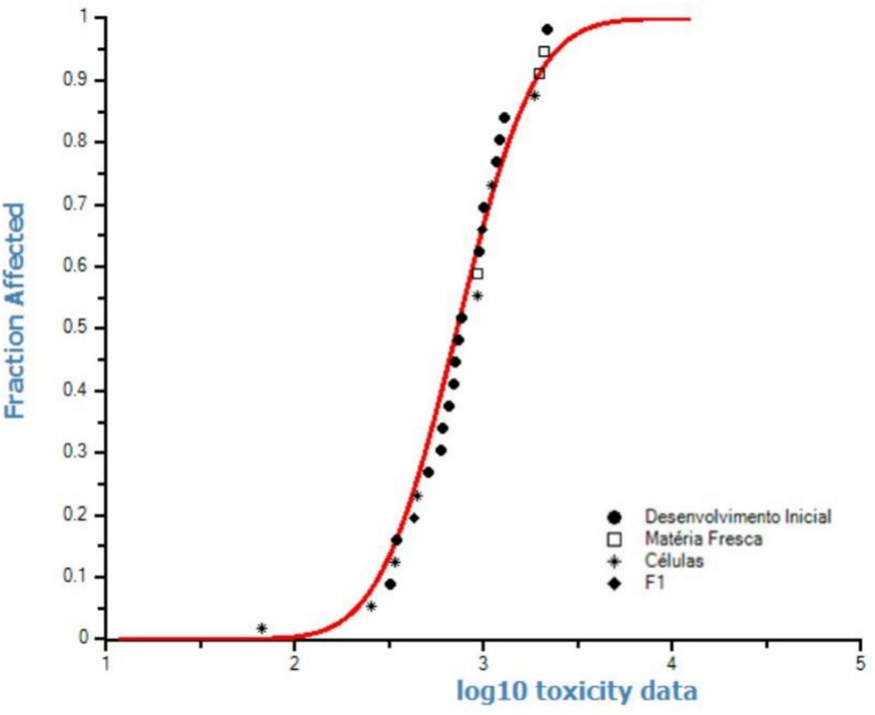
Species sensitivity distribution (SSD) curve and the hazardous concentration that affects at least 5% of the tested parameters (HC_5_) estimated from the EC_50_ values (effective concentration that resulted in 50% inhibition) using the macroscopic and microscopic parameters for the four plant species grown in Latosol and Cambisol soils containing Ce

**Fig. 3:**
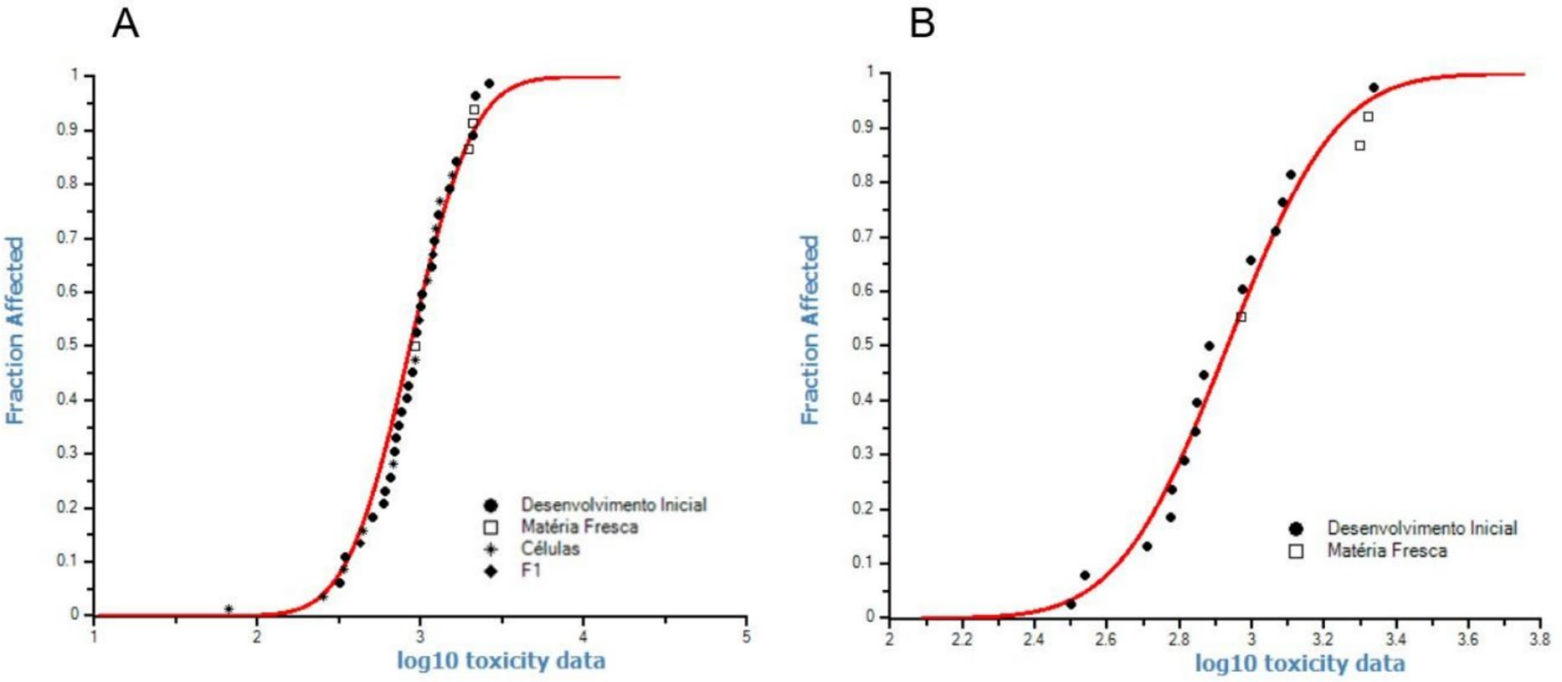
Species sensitivity distribution (SSD) curve and the hazardous concentration that affects at least 5% of the tested parameters (HC_5_) estimated from the EC_50_ values (effective concentration that resulted in 50% inhibition), in A using macroscopic and microscopic parameters for the four species grown in the Latosol, Cambisol, and ATS and in B using only the macroscopic parameters for the four species of plants grown in the Latosol and Cambisol containing Ce

In an ecotoxicological risk assessment, an ecosystem is considered not at risk when 95% of the EC_50_ values of the species in question are protected. For this purpose, with the EC_50_ values, the SSD curves were plotted with the HC_5_ value derived for Ce (Figs. 2 and 3). The HC_5_ values derived for Ce based on the Latosol and Cambisol results are shown in Fig. 2, and the estimated value through the SSD curves was 208.4 mg Ce kg^−1^ of dry soil. Another SSD curve (Fig. 3a) was plotted for the calculation of HC_5,_ including the ATS, and the estimated value was 268.5 mg Ce kg^−1^.

In addition, the HC_5_ values of the Latosol and Cambisol were not used when the microscopic parameters were analysed to determine their influence on the SSD curve, and the HC_5_ value obtained was 344.5 mg Ce kg^−1^ (Fig. 3b).

## Discussion

### Effects of Ce on germination

Studies have shown that at low concentrations, Ce causes positive effects (Espindola et al., 2013; Zhang et al., 2013) on the G% and GSI of seeds (Fashui, 2002). However, there are also reports that at high concentrations, Ce can be harmful to plants, but the mechanism of toxicity is not yet fully known (Moreira et al., 2019; Tyler, 2004).

Fashui (2002) observed that increased germination in rice seeds treated with Ce is associated with an increased respiratory rate and antioxidant system activity. After treating wheat seeds with different doses of Ce, Liu and Liu (1985) noted an acceleration in the activity of α-amylase in the aleuroma layer induced by gibberellin. Such an effect on the enzyme activity resulted in an increase in the germination rate.

In maize and lettuce seeds treated with Ce, the authors did not observe significant differences in the first germination count (Barbieri et al., 2013; Espindola et al., 2013). In these studies, the doses tested were not sufficient to influence germination.

The LOEC/NOEC patterns for %G and GSI indicate that, under the present exposure conditions, these endpoints were generally not sensitive enough to capture Ce phytotoxicity across the tested tropical soils. Among the species evaluated, *L. sativa* showed the clearest response to Ce, whereas *P. vulgaris* and *P. glaucum* were the least responsive and LOEC could not be determined in most soil × endpoint combinations. Overall, the absence of consistent effects on germination corroborates greenhouse evidence reported by Moreira et al. (2019), who tested multiple crops in Ce-spiked tropical soils following OECD 208 and likewise observed limited responsiveness of germination endpoints.

### Effects of Ce on initial seedling development

The positive effects of low concentrations of Ce on root growth (Diatloff et al., 2008; Zhang et al., 2013) and plant height (PH; He & Loh, 2000) have already been reported (Espindola et al., 2013; Jiang et al., 2017; Zhang et al., 2013). However, at high levels, Ce can cause harmful effects and reduce root length (Thomas et al., 2014) and dry weight (Hu et al., 2004).

 The effect of Ce on plants varies according to each species due to the specific physiological, metabolic, and anatomical characteristics of each species, which generate different tolerance mechanisms (Assad et al., 2017; Hameed et al., 2016; Kim et al., 2018; Rizwan et al., 2018; Yang et al., 2017). Therefore, it is important to use monocotyledonous and eudicotyledonous species in ecotoxicological tests, as suggested by the OECD-208 and ISO 11269-2 (ISO, 2013) standards and employed in the present study.

Possibly, the negative effects observed on the growth of the species tested in the three soils may have occurred due to the accumulation of Ce in the cells, promoting a negative effect on cell structure by destroying the plasma membrane (Yang et al., 2015), and due to disturbance in the cell membrane nutrient balance (Pošćić et al., 2017; Xue et al., 2012).

In the present study, decreases in the roots and hypocotyls of *L. sativa*, *A. cepa*, *P. vulgaris* and *P. glaucum* were observed when exposed to higher doses of Ce. However, *L. sativa* was the most sensitive species, while *P. vulgaris* and *P. glaucum* showed lower sensitivity to Ce; such results were possibly due to the tolerance of each species as reported by several authors. In general, for initial seedling development, the EC_50_ and EC_25_ values were lower in the Latosol than in the Cambisol and, finally, in the ATS.

 In ecotoxicological risk assessments, plant development is considered an important and reliable parameter to be evaluated (Moreira et al., 2019; Martins et al., 2019). In the present study, the results obtained for EC_X_ corroborate the length of the roots and hypocotyls and showed them to be responsive to different concentrations of Ce in tropical soils. This soil-dependent sensitivity pattern is consistent with matrix controls on Ce availability and supports the use of early growth endpoints as a more responsive screening tier than germination alone.

### Cell cycle

Cytotoxic and genotoxic effects of Ce in higher plants remain comparatively less explored under realistic soil exposures, as many studies have been conducted in aqueous solutions rather than soil matrices (Kotelnikova et al., 2019). Assessing *A. cepa* directly in Ce-spiked soils is therefore relevant because soil properties can buffer, transform, and partition Ce, potentially decoupling nominal dose from biologically available fractions and modulating cytogenetic outcomes.

 The MI refers to the total number of cells in division during the cell cycle, and changes in this index help to identify the cytotoxicity caused by different organismal exposure to elements (Fernandes et al.^2007^; D'Aquino et al., 2009). Such parameters are important for determining the genotoxic potential of elements (Vieira & Silveira, 2018) present in the soil.

 In cytogenetic analyses performed on *Allium sativum* L. by Xu et al. (2016), it was observed that the MI decreased as Ce doses increased, causing root growth inhibition at doses higher than 100 μM Ce. These authors associated the reduction in MI with the inhibition of root growth. These results were observed in the present study for *A. cepa* seeds grown in the Latosol, in which there was inhibition of root growth and a decreased MI. The decrease in the MI may have occurred due to inhibition of DNA synthesis (SUDHAKAR ET AL., 2001) or blockage of the G2 phase, preventing the cell from entering mitosis (EL-Ghamery et al., 2000) or causing mitotic inhibition of the chemical element (Sharma & Vig, 2012).

 Kotelnikova et al. (2019), also using *A. cepa*, observed this correlation in a test with REE solutions and suggested that in the case of soil tests, this relationship is probably masked by other factors, which may have occurred for the Cambisol and ATS in the present study. By observing the CA values and determining that EC_X_ values for MI were not in the Cambisol and ATS, it was possible to note that although some abnormalities occurred during the cell cycle, these did not prevent the cycle from proceeding.

 Evaluating the frequency of different types of CAs broadens information on the action of potentially genotoxic elements (KOTELNIKOVA et al., 2019). Some of Cas (e.g., adherent chromosomes) are considered severe and may not be repaired by the cell, inducing the formation of CN (Vieira & Silveira, 2018).

 Kotelnikova et al. (2019) showed that CAs and MI were less pronounced in soils compared to those in soils with Ce solutions. Thus, when introduced into the soil, depending on the species, Ce may become less toxic but may remain toxic to plant growth at high concentrations in contaminated soils (Thomas et al., 2014). However, the mechanism of action of cerium in the cell structure and cell cycle is still not well understood (Kotelnikova et al., 2019).

 Based on this information, if CAs become persistent, then they can activate cell death mechanisms (Vieira & Silveira, 2018). The observation of CN, a nuclear alteration, is cytological evidence that the tested compound can induce cell death (Andrade-Vieira et al., 2014). This mechanism of cell death is related to the destruction and subsequent elimination of damaged cells (Danon et al., 2000).

 As shown in the results, it was possible to obtain EC_50_ and EC_25_ values for CN and MCN in all soils tested. These observations demonstrate that Ce at high concentrations was toxic and may be lethal to cells at the highest doses. Such cytogenetic damage cannot be observed in a risk assessment that considers only macroscopic variables, reinforcing the importance of assessments focusing on microscopic variables.

### Micronuclei in the F1 region

The MCN test is the simplest and most reliable *endpoint* for the analysis of mutagenic effects caused by chemical compounds (Vieira & Silveira, 2018). As excess genetic material or DNA damage can be eliminated from the main nucleus in the form of a micronucleus, it is considered an indicator of the maintenance of cellular physiology (Shimizu et al., 2000).

If present in the daughter cells, as observed and calculated by the EC_50_ and EC_25_ values, then MCN are easy to visualize and indicate mutagenic effects because they result from damage that was not repaired or was incorrectly repaired in the meristematic cells (Leme & Marin-Morales, 2009; Vieira & Silveira, 2018). The maintenance of the MCN and the possibility of calculating the ecogenotoxicological indices in the F1 region of the cells corroborate the results of Palmieri et al. (2016), who stated that F1 cells are appropriate for evaluating damage due to chemical compounds.

In the present study, the EC_50_ and EC_25_ values found for the MCN induced by Ce in the three soils may be indicative of mutagenic action. Together, the meristematic and F1-region MCN responses indicate that Ce can exert mutagenic pressure under soil exposure, reinforcing the value of including cytogenetic biomarkers as a complementary line of evidence in screening-level soil hazard evaluations.

### Soil properties, partitioning, and Ce bioavailability

The magnitude of Ce effects differed among soils, consistent with the strong control exerted by soil physicochemical and mineralogical attributes over Ce partitioning between solid phases and soil solution (Farooq et al., 2016). In soils, Ce occurs mainly as Ce(III) and Ce(IV), and its speciation is governed by hydrolysis, complexation, and redox conditions; Ce(IV) tends to form poorly soluble phases and/or strongly sorbed species, whereas Ce(III) can be comparatively more mobile under acidic conditions (Loell et al., 2011; Tyler, 2004).

A key mechanism limiting Ce bioavailability is sorption to reactive mineral surfaces—particularly Fe/Al (oxyhydr)oxides and clay minerals—via inner-sphere complexation and surface precipitation, as well as incorporation into or association with mineral fractions over time (Cao et al., 2001; Wang et al., 2001). Soil organic matter can further modulate Ce behavior through complexation and by providing additional sorption sites, although the direction and magnitude of this effect depend on organic matter quality and competing ligands (Loell et al., 2011).

In this study, the Latosol showed greater toxicity (lower ECx values) than the Cambisol and ATS for most endpoints. This pattern is consistent with matrix controls: lower CEC and lower pH typically favor higher dissolved/available fractions by reducing electrostatic retention and enhancing desorption/solubility of trace elements from mineral surfaces (Cao et al., 2001; Li et al., 2001). The Latosol had the lowest CEC among the tested substrates, which may have reduced Ce retention and increased the fraction accessible to roots, contributing to stronger biological responses. Similar negative relationships between CEC and Ce desorption/availability have been reported for other soil sets and for tropical soils spiked with Ce (Li et al., 2001; Moreira et al., 2019).

Importantly, because post-spiking Ce concentrations and soil-solution speciation were not analytically verified here, these mechanistic interpretations should be viewed as consistent with established geochemical behavior rather than as direct measurements of dissolved Ce. Nonetheless, the soil-dependent effect thresholds observed across macroscopic and cytogenetic endpoints reinforce that Ce hazard cannot be inferred from nominal dose alone without considering matrix-driven partitioning processes.

### Ecotoxicological risk assessment and interpretation of screening thresholds

The SSD-derived HC5 values provided here should be interpreted as screening-level benchmarks derived from a limited set of test species/endpoints and under a specific spiking and exposure design. They are therefore not intended to be universally generalizable to all soils without considering matrix controls on Ce partitioning and bioavailability. The consistent soil-dependent patterns in ECx values indicate that different soils can yield different effective thresholds at the same nominal Ce dose, supporting the use of soil-specific or soil-normalized assessments whenever possible.

Regarding the inclusion of the artificial tropical soil (ATS), the ATS is useful as a standardized substrate to support comparability across studies, but it is not representative of natural mineral assemblages and reactive sorption phases in field soils. In our dataset, inclusion of ATS shifted the SSD outcome and tended to reduce confidence in translating the derived HC5 to natural tropical soils. For this reason, we prioritize the HC5 derived from the two representative Brazilian soils (Latosol and Cambisol) as the most policy-relevant screening threshold for tropical agroecosystems, while retaining ATS-based results as a sensitivity check rather than as a basis for environmental guideline proposals.

 Furthermore, it is believed that the addition of new cytogenetic parameters as additional variables when evaluating the ecological risks of contaminated soils may be relevant for improving the sensitivity of plant tests. Bioassays using microscopic variables for ecotoxicological tests demonstrate great efficiency in the monitoring of environmental pollutants and are important for evaluating genotoxicity and mutagenicity, thus allowing the simultaneous evaluation of multiple mechanisms of action of such agents (Leme & Marin-Morales, 2009).

 When analysing the results obtained in the present study and comparing them with the results of the studies conducted by Moreira et al. (2019) in a greenhouse with the same soils with Ce, it is possible to show the similarity of the results. Even using different species, the HC_5_ values were not substantially different; however, the values in this study were less toxic, probably due to the cytogenetic indices, which detected the damage before it became visible.

This suggests that incorporating cytogenetic biomarkers can change screening decisions by flagging hazard at concentrations where macroscopic growth impairment is not yet evident.

### Practical implications and limitations

This study demonstrates that rapid Petri-dish soil bioassays can support screening-level hazard evaluation of Ce in tropical soils using a combination of macroscopic growth endpoints and cytogenetic biomarkers. The approach is operationally advantageous for prioritization because it requires small soil volumes and short exposure periods compared with greenhouse pot tests (e.g., OECD 208), enabling faster triage of soils, contaminants, and concentration ranges for follow-up evaluation.

At the same time, the present work has important limitations. First, reported Ce doses refer to nominal spiked concentrations; post-spiking analytical verification of total and/or extractable Ce and post-spiking soil pH were not available, which constrains direct interpretation in terms of dissolved Ce and field-equivalent exposure. Second, although the cytogenetic biomarkers improved sensitivity relative to germination endpoints, the battery remains a subset of possible soil-relevant endpoints and does not constitute a validated regulatory protocol by itself. Future studies should incorporate analytical confirmation of exposure (total and bioaccessible fractions), evaluate time-dependent equilibration/aging, and test additional soil types to support soil-normalized thresholds and strengthen transferability across regions.

Despite these constraints, the consistent soil-dependent patterns observed across endpoints support the use of effect-based screening as a complementary line of evidence for soil monitoring programs, particularly when the goal is prioritization rather than definitive site-specific guideline derivation.

## Conclusion

This study provides a rapid screening-level assessment of cerium (Ce) phytotoxicity and cytogenotoxicity in contrasting tropical soils using Petri-dish soil assays. Consistent with our hypothesis, Ce effects were soil-dependent, with stronger responses generally observed in the Latosol than in the Cambisol and the artificial tropical soil, highlighting the role of matrix controls on Ce availability. Incorporating *Allium cepa* cytogenetic biomarkers added a complementary line of evidence, indicating that cellular-level alterations may occur even when germination endpoints are weakly responsive.

The SSD-derived HC5 (208.4 mg Ce kg^−1^) should be interpreted as a screening benchmark for plant protection under the conditions tested, rather than a universal guideline value applicable to all soils. Key limitations include the use of nominal (spiked) Ce concentrations without post-spiking analytical verification (total/bioaccessible Ce and soil-solution speciation), the short exposure duration, and the restricted set of test species and endpoints. Future work should combine effect-based assays with analytical confirmation of exposure and expand soil types and biological models to improve transferability and support higher-tier risk evaluation.

## Supplementary Information

Below is the link to the electronic supplementary material.

Supplementary file1 (PDF 107 KB)

## Declarations

### Data availability

The data of this study are available from the corresponding author, L. F. Andrade-Vieira, upon reasonable request.
