# Supplementary material for: Phytotoxicity and cytogenotoxicity screening of cerium in contrasting tropical soils using rapid Petri-dish assays
Source: Environ Geochem Health. 2026 Feb 23;48(4):189. doi: 10.1007/s10653-026-03073-3 (PMC12929232; doi:10.1007/s10653-026-03073-3)
Supplement: Supplementary file 1 — Supplementary file1 (PDF 107 KB) [file 10653_2026_3073_MOESM1_ESM.pdf]

Supplementary Table S1. Germination (%) and GSI: mean ± SD (n=5) across all Ce doses, per soil and species + Dunnett vs control.

Species	Soil	Endpoint	Control (mean ± SD)	NOEC (mg kg^{-1})	LOEC (mg kg^{-1})	LOEC (mean ± SD)	Highest dose (mg kg^{-1})	Highest (mean ± SD)	n (replicates)
Bean	Artificial soil	Germination (%)	98.00 ± 4.47	709,9	1206.9	82.00 ± 14.83	2051,7	92.00 ± 13.04	5
Bean	Artificial soil	Germination speed index (GSI)	4.80 ± 0.27	709,9	1206.9	3.90 ± 0.65	2051,7	4.00 ± 0.57	5
Bean	Cambisol	Germination (%)	98.00 ± 4.47	2051,7	n.s.	—	2051,7	100.00 ± 0.00	5
Bean	Cambisol	Germination speed index (GSI)	4.50 ± 0.75	2051,7	n.s.	—	2051,7	4.05 ± 0.51	5
Bean	Latosol	Germination (%)	98.00 ± 4.47	2051,7	n.s.	—	2051,7	100.00 ± 0.00	5
Bean	Latosol	Germination speed index (GSI)	5.38 ± 1.38	709,9	1206.9	3.73 ± 1.17	2051,7	4.20 ± 0.50	5
Lettuce	Artificial soil	Germination (%)	97.60 ± 2.19	144,5	245.7	71.20 ± 7.69	2051,7	88.80 ± 5.93	5
Lettuce	Artificial soil	Germination speed index (GSI)	19.01 ± 4.26	0	50.0	8.66 ± 1.78	2051,7	5.28 ± 1.28	5
Lettuce	Cambisol	Germination (%)	95.20 ± 3.35	709,9	1206.9	32.80 ± 13.68	2051,7	12.00 ± 10.20	5
Lettuce	Cambisol	Germination speed index (GSI)	23.20 ± 1.60	0	50.0	7.14 ± 3.72	2051,7	0.08 ± 0.19	5
Lettuce	Latosol	Germination (%)	97.60 ± 3.58	144,5	245.7	59.20 ± 15.85	2051,7	10.40 ± 4.56	5
Lettuce	Latosol	Germination speed index (GSI)	12.11 ± 0.76	0	50.0	4.15 ± 0.92	2051,7	0.15 ± 0.17	5
Millet	Artificial soil	Germination (%)	90.00 ± 7.07	2051,7	n.s.	—	2051,7	78.00 ± 24.90	5
Millet	Artificial soil	Germination speed index (GSI)	8.45 ± 0.45	2051,7	n.s.	—	2051,7	7.20 ± 2.41	5
Millet	Cambisol	Germination (%)	88.00 ± 13.04	2051,7	n.s.	—	2051,7	82.00 ± 14.83	5
Millet	Cambisol	Germination speed index (GSI)	8.60 ± 1.52	2051,7	n.s.	—	2051,7	6.55 ± 1.28	5
Millet	Latosol	Germination (%)	94.00 ± 8.94	50	85.0	72.00 ± 10.95	2051,7	72.00 ± 10.95	5
Millet	Latosol	Germination speed index (GSI)	9.10 ± 0.74	0	50.0	7.20 ± 1.39	2051,7	5.55 ± 1.13	5
Onion	Artificial soil	Germination (%)	85.60 ± 16.64	2051,7	n.s.	—	2051,7	78.40 ± 6.07	5
Onion	Artificial soil	Germination speed index (GSI)	8.45 ± 1.44	245,7	417.6	6.45 ± 1.61	2051,7	7.18 ± 0.92	5
Onion	Cambisol	Germination (%)	24.80 ± 8.20	2051,7	n.s.	—	2051,7	28.00 ± 8.49	5
Onion	Cambisol	Germination speed index (GSI)	1.90 ± 0.63	2051,7	n.s.	—	2051,7	2.08 ± 0.64	5
Onion	Latosol	Germination (%)	31.20 ± 13.08	2051,7	n.s.	—	2051,7	17.60 ± 6.07	5
Onion	Latosol	Germination speed index (GSI)	2.42 ± 1.00	2051,7	n.s.	—	2051,7	1.50 ± 0.76	5

Supplementary Table S2. Seedling growth endpoints (root length, hypocotyl length, etc.) e fresh mass: mean ± SD (n=5) across all doses + (se houver) comparação vs controle.

Species	Soil	Endpoint	Control (mean ± SD)	NOEC (mg kg^{-1})	LOEC (mg kg^{-1})	LOEC (mean ± SD)	Highest dose (mg kg^{-1})	Highest (mean ± SD)	n (replicates)
Bean	Artificial soil	Root length	28.14 ± 3.00	709,9	1206.9	15.67 ± 7.48	2051,7	5.59 ± 0.66	5
Bean	Artificial soil	Hypocotyl length	11.17 ± 1.70	2051,7	n.s.	—	2051,7	7.12 ± 0.47	5
Bean	Artificial soil	Total seedling length	39.31 ± 3.28	1206,9	2051.7	12.71 ± 1.10	2051,7	12.71 ± 1.10	5
Bean	Cambisol	Root length	27.89 ± 3.66	417,6	709.9	19.59 ± 2.80	2051,7	10.69 ± 1.89	5
Bean	Cambisol	Hypocotyl length	11.38 ± 1.72	0	50.0	20.65 ± 4.19	2051,7	10.24 ± 1.26	5
Bean	Cambisol	Total seedling length	39.27 ± 3.45	0	50.0	52.45 ± 10.34	2051,7	20.93 ± 2.95	5
Bean	Latosol	Root length	21.58 ± 7.59	144,5	245.7	29.98 ± 6.78	2051,7	3.50 ± 0.69	5
Bean	Latosol	Hypocotyl length	12.03 ± 3.99	144,5	245.7	17.90 ± 2.74	2051,7	6.21 ± 1.22	5
Bean	Latosol	Total seedling length	33.61 ± 11.48	144,5	245.7	47.89 ± 7.49	2051,7	9.71 ± 1.72	5
Lettuce	Artificial soil	Root length	10.91 ± 0.73	50	85.0	14.90 ± 3.59	2051,7	1.82 ± 0.45	5
Lettuce	Artificial soil	Hypocotyl length	16.25 ± 0.90	417,6	709.9	7.94 ± 2.29	2051,7	0.10 ± 0.23	5
Lettuce	Artificial soil	Total seedling length	27.16 ± 1.13	417,6	709.9	13.84 ± 3.25	2051,7	1.92 ± 0.66	5
Lettuce	Cambisol	Root length	8.87 ± 3.19	709,9	1206.9	0.99 ± 0.45	2051,7	0.37 ± 0.29	5
Lettuce	Cambisol	Hypocotyl length	11.70 ± 4.05	50	85.0	7.28 ± 2.02	2051,7	0.00 ± 0.00	5
Lettuce	Cambisol	Total seedling length	20.57 ± 7.21	417,6	709.9	12.33 ± 2.15	2051,7	0.37 ± 0.29	5
Lettuce	Latosol	Root length	12.73 ± 2.02	0	50.0	8.13 ± 1.20	2051,7	0.22 ± 0.11	5
Lettuce	Latosol	Hypocotyl length	17.42 ± 0.98	0	50.0	12.03 ± 2.54	2051,7	0.00 ± 0.00	5
Lettuce	Latosol	Total seedling length	30.14 ± 1.29	0	50.0	20.16 ± 2.95	2051,7	0.22 ± 0.11	5

Millet	Artificial soil	Root length	40.35 ± 12.43	0	50.0	25.81 ± 3.94	2051,7	4.48 ± 1.83	5
Millet	Artificial soil	Hypocotyl length	17.93 ± 4.41	1206,9	2051.7	10.14 ± 2.91	2051,7	10.14 ± 2.91	5
Millet	Artificial soil	Total seedling length	58.27 ± 16.57	50	85.0	30.24 ± 6.03	2051,7	14.63 ± 4.54	5
Millet	Cambisol	Root length	49.89 ± 14.53	0	50.0	23.00 ± 5.91	2051,7	12.10 ± 1.85	5
Millet	Cambisol	Hypocotyl length	23.36 ± 5.04	1206,9	2051.7	11.91 ± 2.71	2051,7	11.91 ± 2.71	5
Millet	Cambisol	Total seedling length	73.25 ± 19.30	0	50.0	48.12 ± 10.05	2051,7	24.01 ± 4.54	5
Millet	Latosol	Root length	57.94 ± 10.30	0	50.0	19.35 ± 2.76	2051,7	2.80 ± 0.56	5
Millet	Latosol	Hypocotyl length	23.46 ± 2.99	0	50.0	15.17 ± 4.08	2051,7	5.42 ± 1.46	5
Millet	Latosol	Total seedling length	81.40 ± 11.22	0	50.0	34.52 ± 6.27	2051,7	8.22 ± 1.98	5
Onion	Artificial soil	Root length	11.26 ± 3.57	245,7	417.6	5.32 ± 1.38	2051,7	2.21 ± 0.25	5
Onion	Artificial soil	Hypocotyl length	12.65 ± 2.46	50	85.0	6.97 ± 0.92	2051,7	2.95 ± 0.32	5
Onion	Artificial soil	Total seedling length	23.91 ± 5.98	50	85.0	15.34 ± 3.11	2051,7	5.17 ± 0.32	5
Onion	Cambisol	Root length	0.12 ± 0.03	2051,7	n.s.	—	2051,7	0.10 ± 0.03	5
Onion	Cambisol	Hypocotyl length	0.10 ± 0.04	2051,7	n.s.	—	2051,7	0.05 ± 0.04	5
Onion	Cambisol	Total seedling length	0.22 ± 0.06	2051,7	n.s.	—	2051,7	0.15 ± 0.06	5
Onion	Latosol	Root length	1.80 ± 0.85	2051,7	n.s.	—	2051,7	0.67 ± 0.16	5
Onion	Latosol	Hypocotyl length	1.36 ± 0.91	709,9	1206.9	0.12 ± 0.16	2051,7	0.00 ± 0.00	5
Onion	Latosol	Total seedling length	3.16 ± 1.67	709,9	1206.9	0.80 ± 0.40	2051,7	0.67 ± 0.16	5

Supplementary Table S3. Cytogenetic endpoints (meristematic phases/MI; chromosomal and nuclear alterations; MCN in F1): consolidado em tabela (mesmo que venha da dissertação), com nota de que os dados brutos

Soil	Dose (mg kg−1)	Prophase (mean±SD)	Metaphase (mean±SD)	Anaphase (mean±SD)	Telophase (mean±SD)	Mitotic index, MI (mean±SD)
Latosol	0	32.20 ± 16.64	19.40 ± 2.48	8.20 ± 5.04	2.40 ± 0.72	6.22 ± 1.69
Latosol	50	75.80 ± 10.96	28.00 ± 2.00	10.60 ± 3.68	6.20 ± 1.84	12.08 ± 1.02
Latosol	85	14.60 ± 7.36	14.20 ± 4.24	9.80 ± 4.88	4.00 ± 0.80	4.26 ± 1.19
Latosol	144,5	46.20 ± 14.16	27.00 ± 10.80	14.60 ± 8.96	4.40 ± 1.12	9.22 ± 2.66
Latosol	245,7	44.80 ± 11.76	18.00 ± 8.80	11.80 ± 4.96	1.80 ± 0.96	7.64 ± 2.24
Latosol	417,6	50.00 ± 8.00	31.00 ± 10.00	14.80 ± 2.72	5.80 ± 4.16	10.16 ± 1.58
Latosol	709,9	39.20 ± 6.56	17.80 ± 4.88	7.80 ± 4.56	3.40 ± 4.24	6.82 ± 1.49
Latosol	1206,9	30.80 ± 12.16	12.60 ± 5.92	7.60 ± 2.88	6.40 ± 2.08	5.74 ± 2.16
Latosol	2051,7	6.40 ± 0.88	2.20 ± 3.12	1.60 ± 2.56	0.20 ± 0.32	1.04 ± 0.66
Cambisol	0	33.60 ± 17.92	24.80 ± 6.72	4.80 ± 2.64	0.80 ± 0.96	6.32 ± 1.54
Cambisol	50	25.40 ± 8.24	21.60 ± 5.68	8.20 ± 3.52	2.60 ± 1.28	5.75 ± 1.49
Cambisol	85	31.40 ± 8.64	18.80 ± 4.08	6.40 ± 1.68	5.80 ± 1.76	6.21 ± 1.00
Cambisol	144,5	27.20 ± 17.04	15.80 ± 7.36	8.60 ± 3.28	6.00 ± 2.80	5.71 ± 1.82
Cambisol	245,7	20.00 ± 5.20	18.40 ± 8.32	5.80 ± 2.56	4.80 ± 2.64	4.88 ± 1.06
Cambisol	417,6	22.20 ± 6.64	18.00 ± 4.80	3.40 ± 1.28	4.40 ± 1.68	3.79 ± 1.71
Cambisol	709,9	27.60 ± 9.12	11.40 ± 4.24	3.60 ± 1.92	1.80 ± 2.16	4.41 ± 1.63
Cambisol	1206,9	26.60 ± 5.28	12.20 ± 5.04	7.20 ± 2.24	2.80 ± 2.56	4.86 ± 1.01
Cambisol	2051,7	28.00 ± 11.60	11.80 ± 4.56	7.40 ± 4.48	4.40 ± 2.72	5.13 ± 1.69
Artificial soil	0	23.00 ± 8.80	12.60 ± 3.12	13.40 ± 5.28	3.40 ± 2.48	5.24 ± 1.87
Artificial soil	50	15.60 ± 5.68	12.20 ± 5.84	7.20 ± 4.64	3.00 ± 1.60	3.80 ± 0.92
Artificial soil	85	21.00 ± 8.56	12.00 ± 4.32	14.00 ± 4.08	3.00 ± 1.04	5.30 ± 1.74
Artificial soil	144,5	13.40 ± 3.68	13.60 ± 4.32	10.80 ± 3.76	4.80 ± 3.36	4.26 ± 0.76
Artificial soil	245,7	24.20 ± 9.12	21.60 ± 9.92	17.80 ± 8.24	4.00 ± 2.40	6.76 ± 2.41
Artificial soil	417,6	27.80 ± 6.96	16.80 ± 11.76	12.60 ± 4.88	3.40 ± 1.28	6.06 ± 1.83
Artificial soil	709,9	25.20 ± 5.36	17.60 ± 6.08	11.40 ± 6.88	2.80 ± 1.04	5.70 ± 1.08

Artificial soil	1206,9	22.00 ± 6.40	15.80 ± 5.76	13.20 ± 6.64	3.60 ± 1.92	5.46 ± 1.15
Artificial soil	2051,7	22.60 ± 9.76	14.20 ± 6.24	6.60 ± 4.56	2.20 ± 1.52	4.56 ± 1.98

Supplementary Table S4. Assumption tests: Shapiro–Wilk + Levene (p-values e pass/fail), por soil × species × endpoint.

Soil	Species	Endpoint	Shapiro_p	Levene_p	Normality	Homoscedasticity	Decision
Latossolo	Letuce	Germination (%)	0,0082	0,1051	fail	pass	Prefer CR modeling / interpret with caution
Latossolo	Letuce	GSI	0,8206	0,4107	pass	pass	Parametric (ANOVA/Dunnett) eligible
Latossolo	Letuce	Root length	0,0391	0,0735	fail	pass	Prefer CR modeling / interpret with caution
Latossolo	Letuce	Hypocotyl length	0,1592	0,0558	pass	pass	Parametric (ANOVA/Dunnett) eligible
Latossolo	Cebola	Germination (%)	0,8233	0,7435	pass	pass	Parametric (ANOVA/Dunnett) eligible
Latossolo	Onion	GSI	0,9122	0,9692	pass	pass	Parametric (ANOVA/Dunnett) eligible
Latossolo	Onion	Root length	0,2731	0,3019	pass	pass	Parametric (ANOVA/Dunnett) eligible
Latossolo	Onion	Hypocotyl length	0,1933	0,1083	pass	pass	Parametric (ANOVA/Dunnett) eligible
Latossolo	Milheto	Germination (%)	0,4335	0,968	pass	pass	Parametric (ANOVA/Dunnett) eligible
Latossolo	Millet	GSI	0,2488	0,8035	pass	pass	Parametric (ANOVA/Dunnett) eligible
Latossolo	Millet	Root length	0,0923	0,1749	pass	pass	Parametric (ANOVA/Dunnett) eligible
Latossolo	Millet	Hypocotyl length	0,9569	0,6787	pass	pass	Parametric (ANOVA/Dunnett) eligible
Latossolo	Common bean	Germination (%)	0	0,1883	fail	pass	Prefer CR modeling / interpret with caution
Latossolo	Common bean	GSI	0,0964	0,3262	pass	pass	Parametric (ANOVA/Dunnett) eligible
Latossolo	Common bean	Root length	0,0517	0,0135	pass	fail	Prefer CR modeling / interpret with caution
Latossolo	Common bean	Hypocotyl length	0,1664	0,8256	pass	pass	Parametric (ANOVA/Dunnett) eligible
Cambissol	Letuce	Germination (%)	0,0104	0,169	fail	pass	Prefer CR modeling / interpret with caution
Cambissol	Letuce	GSI	0,0193	0,1035	fail	pass	Prefer CR modeling / interpret with caution
Cambissol	Letuce	Root length	0,4855	0,2237	pass	pass	Parametric (ANOVA/Dunnett) eligible
Cambissol	Letuce	Hypocotyl length	0,9795	0,2445	pass	pass	Parametric (ANOVA/Dunnett) eligible
Cambissol	Onion	Germination (%)	0,4118	0,6074	pass	pass	Parametric (ANOVA/Dunnett) eligible
Cambissol	Onion	GSI	0,8964	0,4932	pass	pass	Parametric (ANOVA/Dunnett) eligible
Cambissol	Onion	Root length	0,6463	0,9166	pass	pass	Parametric (ANOVA/Dunnett) eligible
Cambissol	Onion	Hypocotyl length	0,3592	0,2744	pass	pass	Parametric (ANOVA/Dunnett) eligible
Cambissol	Millet	Germination (%)	0,1193	0,6007	pass	pass	Parametric (ANOVA/Dunnett) eligible
Cambissol	Millet	GSI	0,6778	0,7046	pass	pass	Parametric (ANOVA/Dunnett) eligible
Cambissol	Millet	Root length	0,6801	0,1786	pass	pass	Parametric (ANOVA/Dunnett) eligible
Cambissol	Millet	Hypocotyl length	0,8694	0,8716	pass	pass	Parametric (ANOVA/Dunnett) eligible
Cambissol	Common bean	Germination (%)	0	0,7672	fail	pass	Prefer CR modeling / interpret with caution
Cambissol	Common bean	GSI	0,1189	0,8974	pass	pass	Parametric (ANOVA/Dunnett) eligible
Cambissol	Common bean	Root length	0,2676	0,522	pass	pass	Parametric (ANOVA/Dunnett) eligible
Cambissol	Common bean	Hypocotyl length	0,3174	0,1519	pass	pass	Parametric (ANOVA/Dunnett) eligible
Artificial	Letuce	Germination (%)	0,1761	0,8534	pass	pass	Parametric (ANOVA/Dunnett) eligible
Artificial	Letuce	GSI	0,6998	0,9311	pass	pass	Parametric (ANOVA/Dunnett) eligible
Artificial	Letuce	Root length	0,0304	0,073	fail	pass	Prefer CR modeling / interpret with caution
Artificial	Letuce	Hypocotyl length	0,1767	0,0439	pass	fail	Prefer CR modeling / interpret with caution
Artificial	Onion	Germination (%)	0,8478	0,4242	pass	pass	Parametric (ANOVA/Dunnett) eligible
Artificial	Onion	GSI	0,8618	0,4737	pass	pass	Parametric (ANOVA/Dunnett) eligible
Artificial	Onion	Root length	0,5975	0,4624	pass	pass	Parametric (ANOVA/Dunnett) eligible
Artificial	Onion	Hypocotyl length	0,1507	0,12	pass	pass	Parametric (ANOVA/Dunnett) eligible
Artificial	Millet	Germination (%)	0,3238	0,3978	pass	pass	Parametric (ANOVA/Dunnett) eligible
Artificial	Millet	GSI	0,8823	0,5628	pass	pass	Parametric (ANOVA/Dunnett) eligible

Artificial	Millet	Root length	0,2454	0,0668	pass	pass	Parametric (ANOVA/Dunnett) eligible
Artificial	Millet	Hypocotyl length	0,8404	0,4969	pass	pass	Parametric (ANOVA/Dunnett) eligible
Artificial	Common bean	Germination (%)	0,0006	0,4786	fail	pass	Prefer CR modeling / interpret with caution
Artificial	Common bean	GSI	0,0282	0,5491	fail	pass	Prefer CR modeling / interpret with caution
Artificial	Common bean	Root length	0,556	0,3684	pass	pass	Parametric (ANOVA/Dunnett) eligible
Artificial	Common bean	Hypocotyl length	0,6958	0,4811	pass	pass	Parametric (ANOVA/Dunnett) eligible
